# Fetal thymus graft enables recovery from age-related hearing loss and expansion of CD4-Positive T cells expressing IL-1 receptor type 2 and regulatory T Cells

**DOI:** 10.1186/s12979-015-0053-9

**Published:** 2015-12-15

**Authors:** Hiroshi Iwai, Muneo Inaba

**Affiliations:** Department of Otolaryngology, Takii Hospital, Kansai Medical University, Moriguchi, Osaka Japan; First Department of Medicine, Hirakata Hospital, Kansai Medical University, Hirakata, Osaka Japan

**Keywords:** Age-related hearing loss, Spiral ganglion degeneration, Fetal thymus graft, Interleukin 1 receptor type 2, Regulatory T cell, CD4^+^ T cell

## Abstract

**Background:**

Accumulating evidence has indicated the relationship between the systemic immune system and the central nervous system including the inner ear.

**Results:**

We have shown that age-related developments of T-cell dysfunction, hearing loss, and degeneration of cochlear spiral ganglion (SG) neurons observed in 6-month-old mice were recovered in 12 months old mice which previously given fetal thymus transplants twice. We have also demonstrated that CD4^+^ T cells expressing interleukin 1 receptor type 2 (IL-1R2) and naturally occurring regulatory T cells (nTregs), which expanded in aged 12-month-old mice, were reduced in the thymus-grafted mice of the same age.

**Conclusion:**

It is conceivable that the rejuvenation of systemic immune function by fetal thymus grafts contributes not only to the activation of cellular immunity but also to the decrease of IL-1R2^+^ CD4^+^ T cells or nTregs, which cells accelerate both age-related hearing loss (AHL) and neurodegeneration of the cochlear neurons. Further studies on the interactions among IL-1R2 expression on CD4^+^ T cells, Tregs, and neuronal cells and also on the relationships between fetal thymus grafting and the rejuvenation of systemic immunity should be designed in order to advance towards therapeutic effects on neurosenescence, including AHL.

## Background

Hearing loss has a substantial impact on quality of life via impaired communication and can lead to social isolation, poor psychosocial functioning, reduced physical wellbeing, and unemployment [1, 2]. Age-related hearing loss (AHL), also known as presbycusis, is a universal feature of mammalian aging and involves genetic, cellular, and systemic-level changes in the auditory part of the inner ear, namely, the cochlea, and the part of the brain used for hearing, namely, the central auditory pathway [3]. Although AHL is rapidly increasing in incidence and the third most common chronic medical condition of the aged, affecting about half of the population over 75 years old [1], no strategy has been developed for the prevention and treatment of this neurodegenerative disease.

Recent research in gerontology has shown that inflammaging, a state of chronic systematic inflammation associated with age, is a consequence of immunosenescence, the aging of the immune system, and contributes to the aging process and the development of age-related disabilities and diseases including AHL [4]. Type II diabetes and cardiovascular disease associated with inflammaging have been identified as being linked to AHL severity [5, 6]. Verschuur et al. [4] recently indicated that chronic inflammation represented by the white blood cell count is strongly associated with a worsening of AHL among community-dwelling adults aged over 75 years. Local inner ear immunity is part of the overall systemic response and can induce cochlear degeneration and hearing loss [3, 7–9].

It is widely accepted that immune surveillance results in immune and inflammatory responses in the central nervous system (CNS) including neurons by the infiltration of circulating immune cells and the activation of resident cells such as microglial cells, despite the blood–brain barrier [10, 11]. Interleukin (IL)-1 has been particularly implicated in neurodegeneration [10] and is controlled mainly by interleukin-1 receptor type 1 (IL-1R1) to transduce signals, especially IL-1β and interleukin 1 receptor type 2 (IL-1R2), to diminish IL-1 without any transduction of binding signals of IL-1 [10, 12]. IL-1 receptors interact with IL-1 to modulate the functions of leukocytes including CD4^+^ T cells, all cell types of the brain [12], and spiral ganglion (SG) neurons [13]. Naturally occurring regulatory T cells (nTregs) among regulatory T cells (Tregs) accumulate with advanced age, in spite of thymic involution leading to a dwindling thymic T-cell population and inducible regulatory T cells (iTregs), and promote tissue degeneration and senescence-associated inflammation, as well as the disturbance of immune activation against tumors and pathogens [14]. Depletion of Tregs was also shown to improve neural survival after mechanical injury significantly in an animal model [15].

Among the animal models of AHL, senescence-associated mouse type 1 (SAMP1), a murine inbred strain with a genetic background of AKR mice [16–18], shows the early occurrence of thymic involution and accelerated dysfunction of immunocompetent cells, particularly T cells [17, 19], followed by accelerated AHL with the degeneration of SG neurons [19].

We previously demonstrated that systematic immune dysfunction causes AHL and SG neuron damage in the cochlea and that retardation and prevention of the onset of both AHL and immunosenescence are observed when the mice are bred under immunologically clean environments [20] and when the mice are transplanted with allogeneic bone marrow cells [9], respectively. We have also found that up-regulation of the expression of IL-1R2 genes in CD4^+^ T cells is associated with age-related developments of hearing loss, SG degeneration, and the dysfunction of cellular immunity, and that the rejuvenation of recipient immunity by the inoculation of young CD4^+^ T cells or a fetal thymus graft leads to down-regulation of the genes in CD4^+^ T cells and reduces these features of senescence [21].

The goals of the current study were to examine the therapeutic effects of fetal thymus grafts on systemic immunosenescence and inner ear neurosenescence once they have already occurred and also to clarify the anti-aging mechanisms of Tregs, IL-1 receptors on CD4^+^ T cells, and auditory neurons in the cochlea using SAMP1 mice.

## Results

### Acceptance of syngeneic thymic tissue grafted under the renal capsule in SAMP1 mice aged 12 months old

**Fig. 1:**
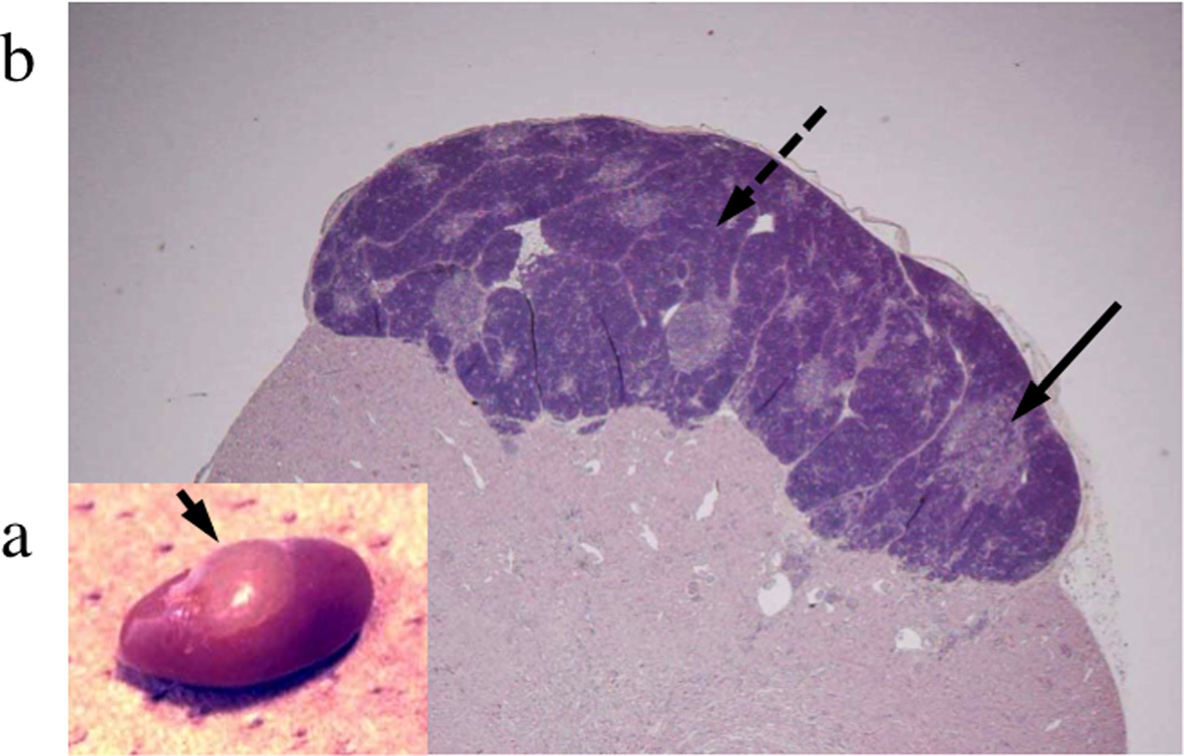
Microscopic and histopathological findings of syngeneic thymic tissue grafted under the renal capsule. **a** Representative microscopic findings. The graft (arrow) was observed under the renal capsule. **b** Representative histopathological findings. H&E staining (original magnification × 10). The graft exhibited acceptance and preservation of the features of thymic tissue consisting of cortex (a plain arrow) and medulla (a dotted arrow). *n* = 10 of bilateral sides of the kidney

Because SAMP1 shows AHL from 5 months old [9], the first thymus transplantation underwent at 6 months old. All thymus grafts under the renal capsule of 10 SAMP1 mice (Group D) showed acceptance and preservation of the thymus structure consisting of cortex and medulla at 12 months old, as shown in Fig. 1.

### Recovery from age-related development of T-cell dysfunction after grafting of the fetal thymus

**Fig. 2:**
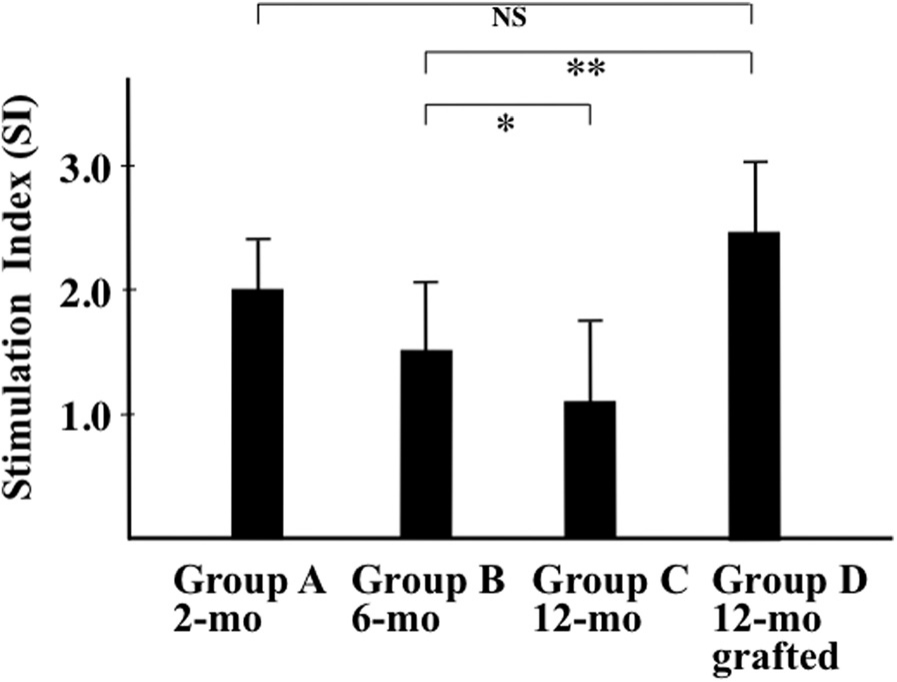
Therapeutic effects of the fetal thymus graft on cellular immunity. To evaluate cellular immunity, the T-cell mitogen (Con A) response was evaluated by MTT assay. Absorbance at 570 nm was determined on a scanning multiwell spectrophotometer. Data are shown at stimulation index (SI), calculated as the ratio of Con A-stimulated to unstimulated T cells. Age-related decline of T-cell functions observed from untreated SAMP1 mice aged 6 months (Group B) to those aged 12 months old (Group C) (*p < 0.05) was recovered in thymus-grafted SAMP1 mice aged 12 months old (Group D, **p < 0.001). There was no significant difference in the Con A responses between untreated SAMP1 aged 2 months old (Group A) and thymus-grafted Group D mice. Experiments were run in triplicate. NS: no significance. *n* = 3–4/group (mean ± SD)

To evaluate cellular immunity, the T-cell mitogen (concanavalin A, Con A) response was evaluated by MTT assay. Although untreated SAMP1 mice showed the development of T-cell dysfunction with aging in Groups B (6 months old) and C (12 months old), Group D, which was 12 months old and had been transplanted with fetal thymi twice at 6 and 8.5 months old, revealed functional recovery with significantly higher proliferative activity than Group B of 6 months old and no significant differences compared with Group A (2 months old) (Fig. 2). Furthermore, to determine the major T cell population in the spleen of the thymus-grafted mice, surface markers of splenic T cells, gated as CD3^+^ cells, were analyzed by flow cytometry. Although there was no significant difference of the frequencies of CD8^+^CD3^+^ T cells between Groups C and D, the frequency of CD4^+^CD3^+^ T cells in Group D was significantly higher than those in Group C (*p* < 0.002) as shown in Table 1, suggesting that the increased T cell proliferative activity of Group D over Group C (Fig. 2) could be the results of increased number of CD4^+^ T cells.

**Table 1:** Evaluation of the major T cell population

Groups	CD4^+^CD3^+^ (%)	CD8^+^CD3^+^ (%)
C (untreated mice of 12 months old)	10.4 ± 0.08	3.85 ± 0.21
D (thymus-grafted mice of 12 months old)	12.9 ± 0.26^*^	3.86 ± 0.36^a^

To evaluate the major T cell population, CD4^+^CD3^+^ T cells and CD8^+^CD3^+^ T cells were analyzed by flow cytometry after the staining with FITC-anti-mouse CD4 mAb plus PE-anti-mouse CD3 mAb, or FITC-anti-mouse CD8 mAb plus PE-anti-mouse CD3 mAb

^*^The frequency of CD4^+^CD3^+^T cells in Group D was significantly higher than those in Group C (*p* < 0.002)

^a^There was no significant difference of the frequencies of CD8^+^ CD3^+^T cells between Groups C and D. All experiments were in triplicate *n* = 3/group (mean ± SD)

### Recovery from auditory dysfunction after grafting of the fetal thymus

**Fig. 3:**
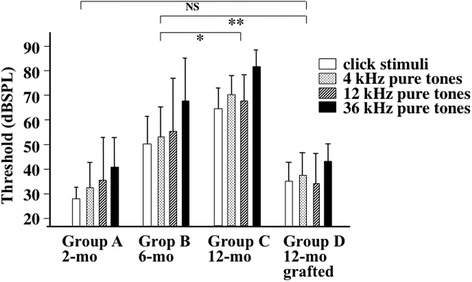
Therapeutic effects of the fetal thymus graft on age-related hearing loss. The auditory functions of SAMP1 mice were examined using ABR at click stimuli and pure tones of 4, 12, and 36 kHz. The development of AHL observed in untreated SAMP1 mice (*p < 0.05–0.005 between Group B aged 6 months old and Group C aged 12 months old) was canceled in thymus-grafted Group D aged 12 months old (**p < 0.01–0.0005 between Groups B and D). The thresholds of Group D showed no significant difference from those of Group A consisting of 2-month-old SAMP1 mice (*p* > 0.05 between Groups A and D). NS: no significance. *n* = 10/group (mean ± SD)

The auditory functions of SAMP1 mice were examined using auditory brain stem response (ABR) at click stimuli and pure tones of 4, 12, and 36 kHz. While untreated SAMP1 mice revealed deterioration of AHL in Groups B (6 months old) and C (12 months old) as determined by ABR using those sounds, thymus-grafted Group D aged 12 months old revealed significant improvement of the auditory response compared with Group B aged 6 months old and no significant differences from the younger Group A (2 months old) (Fig. 3).

### Reduction of degeneration of SG cells of the cochlea by the fetal thymus graft

**Fig. 4:**
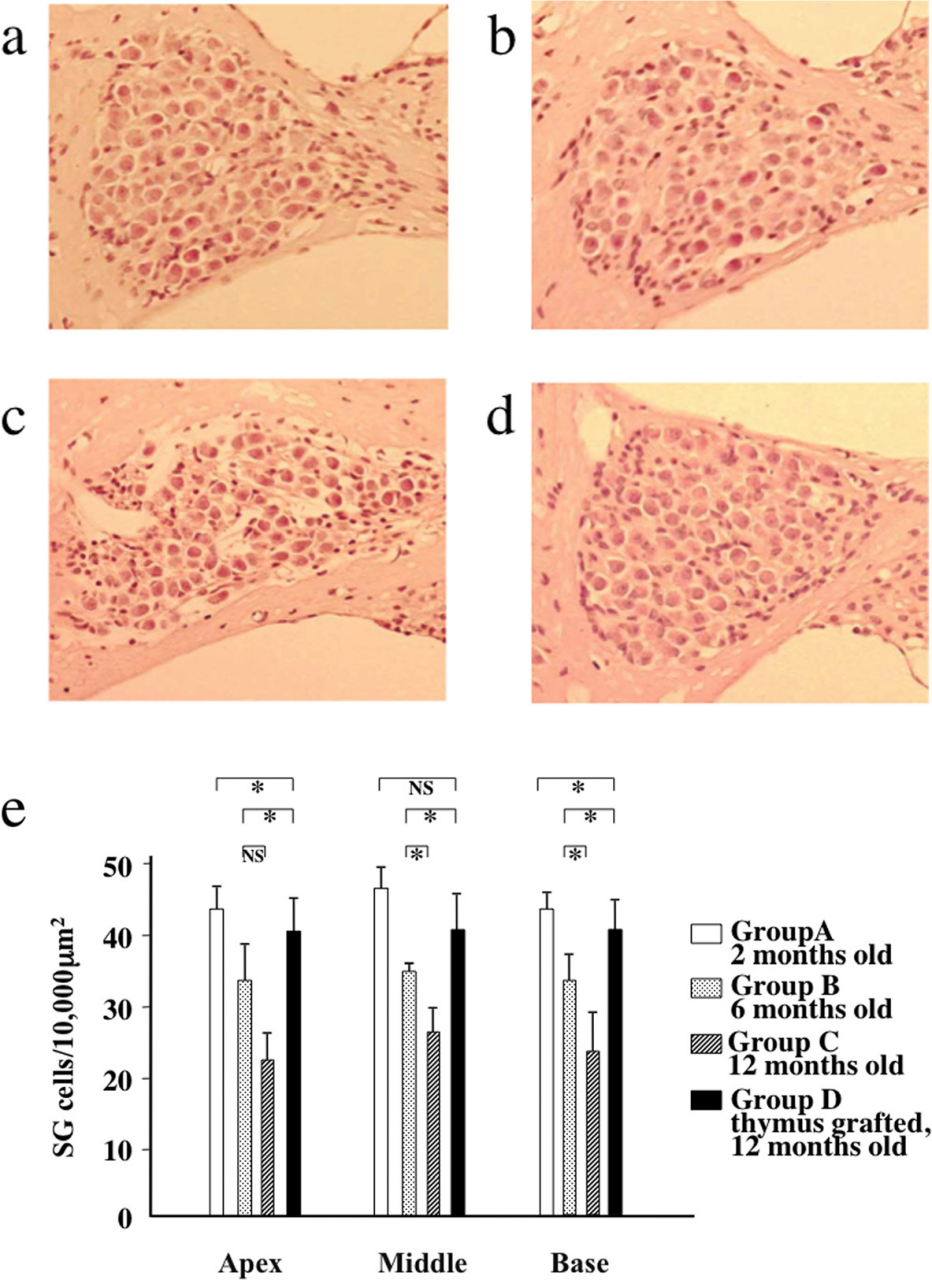
Anti-neurodegenerative effects of the fetal thymus graft on SG cells of the cochlea. SG cells in Groups A (**a**), B (**b**), C (**c**), and D (**d**) were histopathologically examined. Representative findings. H&E staining (original magnification x100). Several cells showed degeneration in the spiral ganglion of Group B aged 6 months old and the development of these findings in Group C aged 12 months old as aging. On the other hand, thymus-grafted Group D revealed no remarkable changes in SG cells, compared with Group A aged 2 months old. **e**) To evaluate degeneration of the auditory neurons, the nuclei of SG cells in the apical, middle, or basal turns of the cochlea were quantified. Although no significant difference of density of the nuclei/10,000 μm^2^ was observed between Group B aged 6 months old and Group C aged 12 months old at the apical turn (*p* = 0.065), age-related devastation of the density of SGs between Groups B and C was found in the other turns (*p* < 0.005–0.0005), and significant recovery of the density in thymus-grafted Group D in comparison with that in Group B at all turns (*p* < 0.01–0.00005) was observed. The significant recovery of neurodegeneration in Group D was insufficient to reach the level of Group A in the middle turn (*p* = 0.033); however, no significant difference in comparison with Group A was observed in any other turns (*p* > 0.05). NS: no significance. *n* = 5 for Groups A-C and 10 for Group D (mean ± SD)

SG cells in Groups A, B, C, and D were histopathologically examined as shown in Figs. 4a-d. Spiral ganglion cells showed low cell density in Group B aged 6 months old and the development of these findings in Group C aged 12 months old as aging. On the other hand, thymus-grafted Group D revealed no remarkable changes in SG cells, compared with Group A aged 2 months old.

The nuclei of SG cells in the apical, middle, or basal turns of the cochlea were also quantified to evaluate degeneration of the auditory neurons (Fig. 4e). Untreated mice of Groups B and C histopathologically showed age-related developments of degeneration in SGs of the middle and basal turns. On the other hand, thymus-grafted Group D showed significantly higher density of the nuclei than Group B in all turns and did not reveal a significant difference from Group A aged 2 months old in the apical and basal turns.

### Fetal thymus graft suppresses the age-related increase in expression of IL-1R2 and FR4 on splenic CD4^+^ T cells and does not change regulation of IL-1R1

**Fig. 5:**
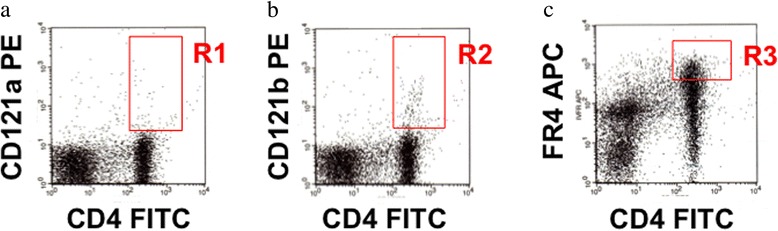
Gating for IL-1R1, IL-1R2, and FR4 on CD4^+^ T cells. Surface markers of IL-1R1 (**a**), IL-1R2 (**b**), and FR4 (**c**) on CD4^+^ T cells were examined using FITC-conjugated anti-mouse CD4 cell mAb, PE-conjugated anti-mouse CD121a (IL-1R1) mAb, PE-conjugated anti-mouse CD121b (IL-1R2) mAb, and biotin-conjugated anti-mouse FR4 (nTreg) mAb plus streptavidin-RED670 with flow cytometry. a) Square R1, b) square R2, and c) square R3 include CD121a^+^ CD4^+^ cells, CD121b^+^ CD4^+^ cells, and FR4^hi^ CD4^+^ cells, respectively

The next step was to examine the effect of the fatal thymus graft on expression of Tregs and IL-1 receptors on CD4^+^ T cells in SAMP1 mice of our accelerated AHL model. Flow cytometric analysis indicated the setting gates (squares of R1-R3) for CD121a (IL-1R1)^+^CD4^+^ cells, CD121b (IL-1R2)^+^CD4^+^ cells, and folate receptor 4 (FR4)^hi^CD4^+^ cells as nTregs (Fig. 5). This FR4 is a functionally essential molecule for Tregs and is constitutively highly expressed on nTregs [22].

Table 2 shows the data from the squares and indicates that IL-1R1 expression on CD4^+^ cells has no difference between any groups without any influence of aging or thymus graft. Although there was no significant difference between Group B aged 6 months old and Group C aged 12 months old in terms of age-related progression in the expression of IL-1R2 and FR4 on CD4^+^cells, Group B showed significant up-regulation of CD121b and FR4 on CD4^+^ cells in comparison with Group A aged 2 months old, via aging. Group D aged 12 months old with thymus graft still showed significant differences from Group A, indicating that the graft could not reveal sufficient effect to down-regulate those surface antigens to the level of Group A; however, Group D showed significant reduction of the receptors of CD121b and FR4 compared with Group B, as a result of fetal thymus transplantation.

**Table 2:** Down-regulation of IL-1R2 and FR4 on splenic CD4^+^ T cells by fetal thvmus graft

Groups	Square R1^a^	Square R2	Square R3
	CD121a^+^ cells (%)	CD121b cells (%)	FR4 ^hi^ cells (%)
	on CD4^+^ cells^*^	on CD4^+^ cells	on CD4^+^ cells
A, 2 months old	0.46 ± 0.07	0.33 ± 0.08	11.02 ± 0.81
B, 6 months old	0.39 ± 0.14	0.83 ± 0.21^**^	23.52 ± 0.80^****^
C, 12 months old	0.46 ± 0.18	1.07 ± 0.13	22.10 ± 1.63
D, 12 months old	0.58 ± 0.28	0.57 ± 0.03^***^	12.85 ± 0.54^*****^
Thymus-grafted			

^a^Squares of R1-R3 are shown in Fig. 5

^*^ *p* > 0.05 between any groups

^**^ *p* < 0005 between Groups A and B. *p* < 0.01 between Groups B and D. *p* > 0.05 between Groups B and C

^***^ *p* < 0.005 between Groups A and D

^****^ *p* < 0.005 between Groups A and B. *p* < 0.005 between Groups B and D. *p* > 0.05 between Groups B and C

^*****^ *p* < 0.01 between Groups A and D. All experiments were run in triplicate *n* = 3–4/group (mean ± SD)

## Discussion

Although cochlear implants can effectively replace the mechanosensory transduction function of lost hair cells by providing direct electrical stimulation of SG neurons in the cochlea, this technique can only be successful when sufficient SG neurons remain [23]. Thus, there is an urgent need to analyze the mechanisms of age-related SG neuron loss with the global increase in the size of the elderly population.

SAMP1 mice used in the current study show accelerated senescence including degeneration of SG neurons, AHL, thymic involution, and cellular immune dysfunction, as well as alopecia, spiral curvature, and shortened lifespan [16, 17, 19]. We previously demonstrated that these mice do not show AHL upon the inoculations of CD4^+^ T cells from a syngeneic donor aged 2 months old twice at a two-month interval or can avoid AHL and SG neuron loss by syngeneic fetal thymus grafting at 2 months old (before the onset of AHL) [21]. These results encouraged us to design the present experiment that attempted to treat the immunosenescence, AHL, and cochlear pathology of SAMP1 mice aged 6 months old (after the onset of AHL) and to obtain insight into the development of Tregs and IL-1 receptors on CD4^+^ T cells, which had been reported to have a relationship to CNS degeneration [10, 12–15].

Figure 1 shows that fetal thymus grafts were accepted, thereby preserving the thymus structure, by syngeneic recipients under the renal capsule, where experimental transplantation of organs is often performed because of the rich vascularity [24]. The age-related developments of T-cell dysfunction, hearing loss, and SG neuron degeneration in the mice aged 6 months old were recovered from at 12 months old after the thymus transplants had been applied twice (Figs. 2, 3 and 4). We previously performed preliminary experiments using a group aged 12 months of sham surgery without fetal thymus grafts and found that there was no difference of T-cell functions and acoustic functions between the sham group and the control group of the same age (data not shown). Thus, cellular supply from grafts of the thymus, which supports hematopoietic progenitor cells migrating from bone marrow and outputs naïve T cells into the systemic immune system [25], enabled recovery from both systemic immunosenescence and cochlear neurosenescence. On the other hand, it is not clear in the current study whether SG neurons, which once showed decline of cell density at 6 month old, increased the number equivalent to that in the mice in 2 months old after fetal thymus transplantation. Because SAMP1 shows hearing loss at 5 months old [9], we have grafted the thymus at 6 (and 8.5) months old. This animal strain shows hearing loss at first, and then reveals progression of neuron loss in SG until 12–14 months old [6]. Six months old is, therefore, considered to be an early phase of degeneration. There may be considered two explanations for recovery of cell density in SG as well as auditory functions comparing thymus-grafted mice aged 12 months with control mice aged 6 months: 1) the neurons once decreased the number at 6 months old due to cell death, however, regenerated and boosted it according to local increase of neurotrophic factors in response to recovery of cellular immunity after thymus graft, and 2) SG started to show dysfunction and decrease of cell density at 6 months due to cell expansion as a consequence of edema and/or accumulation of metabolite in the cytoplasm as a preparatory stage for degeneration, however, survived, reactivated, and shrank the cells by immunological improvement after thymus graft. Further studies are needed to elucidate the effects of cellular immunity modified by the fetal thymus graft on neuronal anti-ageing system. This relationship between systemic immunity and the inner ear is reminiscent of prior findings in our laboratory that revealed that allogeneic transplantation to renew the host immunity by an allogeneic donor treated autoimmune hearing loss and lupus nephritis in MRL/Mp-lpr/lpr mice, a murine model of autoimmune sensorineural hearing loss (SNHL) and systematic lupus erythematosus (SLE) [26].

Rejuvenation of the thymus leads to reconstitution of cellular immunity with functions as good as young cells and better than those of aged mice and humans [27]. On the other hand, thymectomy induces an imbalance between lymphocytes, macrophages, and cytokines, which induces neurotransmitter and neuroendocrine changes through the blood–brain barrier, and memory disturbance in CNS [28]. Both acute and chronic systemic inflammation is associated with an increase in cognitive decline in Alzheimer’s disease [11]. All neuronal cells and locally invading leukocytes including T cells and macrophages can express IL-1 and IL-1R1, which enables them to respond to IL-1 in an autocrine and paracrine manner [10]. Therefore, our next step was to examine surface markers of CD4 T cells that are the center to manage cellular immunity and the relationship with CNS. An increase of IL-1R1 expression and IL-1 production has been reported in aged rodents, suggesting that CD4^+^ T cells from the animals are highly responsive to IL-1R1 stimulation and prepared to differentiate into Th17 cells, which is closely linked to autoimmune diseases [29]. The results, however, indicated that IL-1R1 on CD4^+^ T cells did not show any change in regulation as a consequence of aging or thymus grafting (Fig. 5 and Table 2). We previously indicated that there are no autoimmune mechanisms in AHL in SAMP1 mice because prednisolone administration had no preventive effect against this cochlear dysfunction in the mice [20].

On the other hand, our data demonstrated that the expression of IL-1R2 on the cells was increased by aging and decreased by thymus grafting. IL-1R2 serves as a decoy receptor binding to IL-1, but does not induce signal transduction due to the lack of an intracellular domain [29]. IL-1 receptor antagonist (IL-1Ra) also suppresses the function of IL-1 as a proinflammatory cytokine to prevent the binding of IL-1 and restricts the recruitment of IL-1R accessory protein (IL-1RAcp) [30]. Protective and toxic effects of IL-1 are associated with the exogenous and endogenous concentrations of IL-1 and the localized nature of its actions [10]. The local secretion of IL-1Ra in the cochlea using an adenovirus vector and IL-1Ra cDNA promotes the degeneration of SG cells [13]. Sustained transgenic IL-1 over-expression, as well as *in vitro* exposure to IL-1, contributes to a reduction in amyloid pathology, mediated by enhancement of microglia-dependent plaque degradation, with no evidence for IL-1-associated apoptosis of neurons [31]. After a CNS injury, T cells nonselectively migrate to the site of injury, suggesting that homing T cells, which encounter their relevant antigens at the lesion site, are the ones that contribute to the repair. Such T cells become locally activated to produce cytokines including IL-1 and neurotrophic factors, which are capable of affecting the activity of resident microglia and hence the fate of threatened neurons [14]. Therefore, the increase of expression of IL-1R2 on CD4^+^ T cells in the current study of AHL may be involved in age-related disability, which locally leads to the lack of IL-1 signal transduction, causing cell death.

nTregs express CD4, CD25, and Foxp3 and accumulate with age via thymic involution, while iTreg numbers decrease. The increase of Treg activity generally contributes to autoimmunity in the young and to an impaired anti-tumor response, declining anti-microbial immune responses, and the development of tissue degeneration in the elderly [14]. Yamaguchi et al. [22] reported FR4 is a functionally essential molecule for Tregs and is constitutively highly expressed on nTregs; they also demonstrated that the blockage of FR4 sufficed to deplete CD25^+^CD4^+^ nTregs and that the transfer of FR4^hi^ cell-depleted T-cell suspensions induced autoimmune disease in nude mice that had rejected tumors. In addition, anti-FR4 mAb treatments prevented the development of methylcholanthrene (MCA)-induced sarcoma [32]. We utilized this FR4 as an nTreg marker in the current study and found that the FR4n^hi^ Treg population expanded in the aged mice and shrank in the thymus-grafted mice. Causal links between increased Treg numbers and the incidence of neurodegenerative disease have been suggested since neuron survival was found to be higher in the absence of Tregs in a mouse model of optic nerve injury [15].

The present findings raise the question, which must be answered by subsequent experiments, of which cells are mainly associated with SG degeneration, IL-1R2^+^ CD4^+^ T cells (non-Tregs), Tregs, or IL-1R2^+^ Tregs. While there is no significant difference in suppressive capacity between IL-1^+^ Tregs and IL-1R1^−^ Tregs, Tregs expressing IL-1R2 neutralize IL-1β as a result of TCR activation [33]. Although it is still unclear whether the suppressive effects of activated Tregs contribute to the action of expressed IL-IR2 or the original functions of the Tregs themselves, IL-1R2^+^ CD4^+^ T cells and activated Tregs might be new targets for therapeutic intervention in IL-1-mediated neurodegenerative diseases.

To our knowledge, this study is the first to show that syngeneic transplantation of the thymus can be used to treat AHL and regulate both IL-1R2^+^ CD4^+^ T cells and Tregs.

We evaluated frequencies of splenic CD4^+^ and CD8^+^ T cells of the untreated and the thymus-grafted mice, and the number of CD4^+^ T cells in the grafted mice was more than that of the control mice (Table 1) suggesting that the CD4^+^ T cells were supplied from the grafted thymus and probably contributed to the immune rejuvenation leading to the recovery of Con A responses as shown in Fig. 2. On the other hand, it was not established in the present study with syngeneic thymus grafts whether the CD4^+^ T cells derived from the recipient bone marrow cells and matured through the thymus grafts or from fetal thymus grafts where donor progenitor cells proliferated and seeded recipient peripheral tissues. Because we previously demonstrated that donor immunocompetent cells systemically appeared in SAMP1 mice that had been transplanted allogeneic bone marrow cells at 2 months old before the start of thymic involution, it is likely that the grafted thymus supplied recipient CD4^+^ T cells in the current study.

Recent studies to analyze the development of hearing loss focus not only on the disability in the auditory organ, but also systemic functions affected by metabolic changes, hormones, diet, and the immune system [34]. Our current study using a mouse model suggests at least three strategies to prevent AHL: i) IL-1R2^−^ CD4^+^ T cells or non-Tregs collected at a young age would be preserved and then inoculated several times into autologous recipients that were previously the donors of the cells and then have shown AHL. ii) The use of thymic epithelial cells differentiated from autologous pluripotent stem cells [35]. Grafting of these cells under the renal capsule [24] or injection of these cells into the spleen or the peritoneal cavity [36] might contribute to the treatment of AHL and the rejuvenation of the recipients to decrease IL-1R2^+^ CD4^+^ T cells and Tregs. iii) Finally, the targeting of IL-1R2^+^ CD4^+^ T cells and Tregs with antibodies to treat neurodegeneration could be applied.

## Conclusion

We found in an AHL animal model that the increase of IL-1R2^+^ CD4^+^ T cells and nTregs was associated with the age-related development of T-cell dysfunction and the degeneration of SG in the cochlea, and that fetal thymus grafts treated AHL and reduced neurodegeneration and both IL-1R2^+^ CD4^+^ T cells and nTregs. Therefore, it is conceivable that the rejuvenation of systemic immune function by fetal thymus grafts contributes to the activation of cellular immunity and to the decrease of IL-1R2^+^ CD4^+^ T cells or nTregs, which cause AHL as a consequence of neurodegeneration of the cochlear neurons. Further studies on the interactions among IL-1R2 expression on CD4^+^ T cells, Tregs, and neuronal cells and also on the relationship between the fetal thymus graft and the rejuvenation of systemic immunity should be designed to advance towards therapeutic effects on neurosenescence, including AHL.

## Methods

### Animals and experimental design

SAMP1 were purchased from SLC (Shizuoka, Japan) and maintained under specific pathogen-free conditions in our animal facilities. The care and use of the mice reported in this study were approved by a grant application agency [Grants-in-Aid for Scientific Research (21592170 and 10232638) from the Ministry of Education, Science, Sports and Culture].

We divided SAMP1 mice into 4 groups consisting of 2-month-old SAMP1 mice as Group A, 6-month-old SAMP1 mice as Group B, 12-month-old SAMP1 mice as Group C, and the SAMP1 mice (12 months old) grafted with syngeneic fetal thymus (gestational age of 18–19 days) under the capsule of the left kidney at 6 months old and of the right kidney at 8.5 months old as Group D. These mice were examined for T-cell functions, acoustic functions, and the pathology of the cochlea. CD4^+^ T cells were also analyzed by flow cytometry for their cell surface antigens, especially IL-1 receptors and FR4, which are highly expressed on Foxp3^+^CD25^+^CD4^+^ nTregs [22].

### MTT, 3-(4,5-di-methylthiazol-2-yl)-2,5-diphenyltetrazolium bromide, assay for T-cell function

To examine cellular immune functions, the proliferative responses of splenic T cells were evaluated using the MTT assay in accordance with the manufacturer’s instructions (MTT cell proliferation assay kit; Cayman Chemical Co., Ann Arbor, MI, USA). This assay is based on the capacity of mitochondrial dehydrogenase enzymes in viable cells to convert the yellow water-soluble substrate, MTT, into a dark blue formazan product, which is insoluble in water. A solubilization solution is then added to dissolve the insoluble purple formazan product into a colored solution, which is quantified by its absorbance. Briefly, cells were seeded at a density of 1x10^5^ cells/well in triplicate in 96-well plates with Con A (2.5 mg/ml; Calchem-Behring Corp., San Diego, CA) T-cell mitogen for 2 days, followed by adding an aliquot of MTT solution at 0.5 mg/ml to each well and incubating the plates for 4 h at 37 °C. The medium was then discarded, and the formazan blue formed in the cells was dissolved in dimethyl sulfoxide (DMSO). Absorbance at 570 nm was determined on a scanning multiwell spectrophotometer. Data are expressed at stimulation index (SI), calculated as the mean reading of triplicate wells of antigen (Con A)-stimulated T cells divided by the mean reading of triplicate wells from unstimulated (negative control) wells.

### Analysis of the major T cell population

Surface markers of CD4, CD8, and CD3 (BD Pharmingen, San Diego, CA) were examined with FACScan (Becton Dickinson & Co.) using FITC-conjugated rat anti-mouse CD4 mAb, FITC-conjugated rat anti-mouse CD8 mAb, and PE-conjugated rat anti-mouse CD3 mAb. All experiments were run in triplicate (*n* = 3/group).

### Auditory response

The auditory function of the mice was evaluated by means of ABR, as described previously (Iwai et al., 2003). ABR is an evoked potential measurement of the auditory activity of the cochlea, auditory nerve, and central auditory pathway in the brainstem. Prior to testing, the mice were anesthetized with sodium pentobarbital (60 mg/kg, i.p.) and atropine sulfate (0.5 mg/kg, i.p.). Otoscopic examinations revealed normal tympanic membranes in all mice. Needle electrodes were inserted into the occiput, vertex, and hindpaw (ground). Click stimuli produced by 2 V pulses of alternating polarity of 100 msecond duration and pure tone stimuli of 4 kHz, 12 kHz, and 36 kHz presented for a duration of 5 mseconds with a 1 msecond rise/fall time at a rate of 10 per second were generated using the SigGenRP software package (Tucker-Devis Technologies, Alachua, FL). These sound stimuli were delivered to the left external auditory meatus in a closed acoustic system. The average responses from 1,000 sound stimuli were obtained for each auditory stimulus by reducing the sound intensity until the threshold. The thresholds were verified at least twice and defined as the lowest intensity producing a clearly visible first wave peak, the origin of which is closely connected to spiral ganglion neurons [37] (*n* = 10/group).

### Histopathology

After auditory examination and splenectomy for the mitogen response and flow cytometric analysis, the mice were subjected to intracardiac perfusion with saline followed by 4 % paraformaldehyde phosphate buffer solution and decalcification at 4 °C in 5 % buffered EDTA for 7 days. The temporal bones were sectioned at 4 μm horizontally to the long axis of the cochlea and stained with hematoxylin and eosin (H&E). The sections were randomly selected from these mid-modiolar sections for each animal, and the nuclei of SG neurons in the apical, middle, or basal turn of the cochlea were quantified as previously described [9, 38]. The absolute density value was normalized for 10,000 μm^2^ (*n* = 5 for Groups A-C and 10 for Group D). The grafted tissues of the fetal thymus under the renal capsule of Group D were also evaluated for graft acceptance after staining with H&E (*n* = 10).

### Analysis of expression of IL-1R1, IL-1R2, and FR4 on splenic CD4^+^ T cells

Surface markers of IL-1R1 and IL-1R2 on splenic CD4^+^ T cells were examined with FACScan using FITC-conjugated rat anti-mouse CD4 mAb, PE-conjugated rat anti-mouse IL-1R1 (CD121a) mAb (BD Pharmingen), PE-conjugated rat anti-mouse IL-1R2 (CD121b) mAb (BD Pharmingen), and biotin-conjugated rat anti-mouse FR4 (natural Treg) mAb (Reprocell Co., Tokyo, Japan) plus streptoavidin-Red670 conjugate (SA-RED670; BIBCO, Grand Island, NY). All experiments were run in triplicate (*n* = 3-4/group).

### Statistical analysis

Data are presented as the mean ± SD. Statistical analyses were performed by Student’s *t*-test and ANOVA (StatMateV, Atoms Co. Tokyo, Japan). Statistical significance was accepted at *p* < 0.05.

## Abbreviations

AHL: Age-related hearing loss
CNS: Central nervous system
FR4: Folate receptor 4.
IL-1: Interleukin 1
IL-1R1: Interleukin 1 receptor type I
IL-1R2: Interleukin 1 receptor type II
IL-1Ra: Interleukin 1 receptor type I antagonist
IL-1RAcp: IL-1R accessory protein
iTregs: Inducible regulatory T cells
nTregs: Naturally occurring regulatory T cells
SAMP1: Senescence-associated mouse type 1
SG: Spiral ganglion
Tregs: Regulatory T cells
